# Assessment of Biomedical Waste Management in Health Facilities of Uttar Pradesh: An Observational Study

**DOI:** 10.7759/cureus.20098

**Published:** 2021-12-02

**Authors:** Anand M Dixit, Priyanka Bansal, Pankaj Jain, Prashant K Bajpai, Rama S Rath, Pradip Kharya

**Affiliations:** 1 Department of Community Medicine & Family Medicine, All India Institute of Medical Sciences, Gorakhpur, Gorakhpur, IND; 2 Epidemiology and Public Health, Uttrakhand Health Services, Uttarakhand, IND; 3 Community Medicine, Uttar Pradesh University of Medical Sciences, Saifai, IND

**Keywords:** india, health policy, waste hazards, health care facilities, biomedical waste management

## Abstract

Background

Biomedical waste management has recently emerged as an issue of major concern for every health facility and healthcare provider due to human and environmental hazards. As per government guidelines, every health facility, either large medical institutes or small clinics, should ensure appropriate biomedical waste management at their facilities level.

Objective

To assess biomedical waste management in various health care facilities of Etawah district.

Methodology

It was a facility-based cross-sectional assessment that included government and private health facilities. The selection of facilities was done based on a simple random sampling method. All the people in charge of concerned health care facilities were interviewed to know the current biomedical waste management situation concerning health facilities and the problems they face in biomedical waste management. Health care professionals' knowledge was also assessed.

Results

A total of 56 health care facilities (HCFs) from both government and private sectors were selected. Biomedical waste guidelines are mainly available at tertiary care centers (93%) and secondary care centers (51.5%). Awareness among doctors related to hazards and prevention of hazards (<0.001), knowledge of unused sharps (0.048), contact with a blood-related product (0.003), hazardous waste (<0.001), and need for training (<0.001) are statistically significant with respect to nurses.

Conclusions

Government of India guidelines on biomedical waste management (BMW) are in place, but the use of guidelines currently is not up to the mark or at a satisfactory level. Spreading awareness of the BMW guidelines and their strict implementation is the need of the hour.

## Introduction

Biomedical waste management has recently emerged as an issue of major concern for health care facilities, either government or private, and human safety, environmental, and law enforcement agencies. Biomedical waste (BMW) is deﬁned as "any waste generated during diagnosis, treatment, or immunization of human beings or animals or in research activity pertaining to or in the production or testing of biological or in health camps" [1]. Biomedical waste management is a requirement for every health facility for ensuring human safety and environmental sustainability. As per government guidelines, every health facility, large medical institute, or small clinic must ensure appropriate biomedical waste management. A health care facility (HCF) means a place where diagnosis, treatment, or immunization of human beings is provided irrespective of type and size of the health treatment system and research activity pertaining thereto. Government healthcare facilities include district hospitals, sub-divisional hospitals, community health centers, primary health centers, and sub-centers and private facilities, which include large corporate hospitals to small clinics [2]. An HCF is also a source of nosocomial infection, diseases, and adverse environmental impact [3].

Many countries lack government rules regarding BWM. India was one of the first countries to have and implement biomedical waste management (BMWM) rules [4]. The Ministry of Environment and Forest notified the "Bio-medical Waste Management and Handling Rules" in July 1998 (later amended in 2003, 2011, and now in 2016) under the Environment Protection Act, 1986 [5]. Even after a decade of its implementation in India, hospitals have not achieved the desired standards for BMWM practices [6-7].

The Ministry of Environment, Forest, and Climate Change published the latest guidelines on March 28, 2016, and these rules may be called BMWM Rules 2016. These hazardous wastes are theoretically risky because they may be unaffected by treatment and possess high pathogenicity or the ability to cause disease [8]. Biomedical waste rules 2016 - Schedule 1 describes BMW categories in color coding and treatment options. Schedule 2 describes standards for the treatment of biomedical waste. Schedule 3 describes a list of prescribed authorities and the corresponding duties, and Schedule 4 describes labels for biomedical waste bags or containers [1].

Although various studies had been carried out on biomedical waste management nationwide, most studies were conducted in advanced health care centers and tertiary care centers. So, there was an urgent need to assess awareness practices and problems faced in dealing with BMW at all levels of HCFs, including large health institutes to a smaller clinic. So, efforts have been made to study biomedical waste management in all health care centers from the grass-root (sub-center) level to the topmost (medical college, district hospital) level in the private and government systems of Etawah district. The objective of the study was to assess biomedical waste management in various healthcare facilities of Etawah district.

## Materials and methods

This was a facility-based cross-sectional evaluation conducted at all levels of health care facilities, three-tier government health facilities, and private health facilities. The study was conducted from 2017 to 2018.

Operational definitions

Health Care Facility (HCF)

A health care facility means a place where the diagnosis, treatment, or immunization of human beings is provided irrespective of the type and size of the health treatment system and the research activity pertaining thereto. These health care facilities include medical colleges, district hospitals, community health centers, primary health centers and sub-centers, private hospitals, and private clinics.

Government Health Care Facilities

All health care facilities that are managed and funded by the state or central government.

Private Hospital

An HCF that is managed by an individual or group of doctors with OPD and IPD facilities. Hospitals with a minimum five-bedded capacity were studied.

Private Clinic

These were types of HCF where only OPD was undertaken.

Selection of facilities

**Figure 1:**
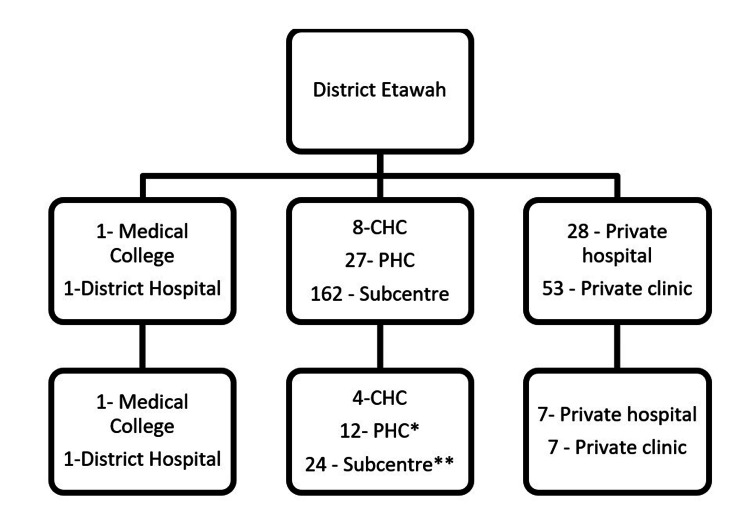
Distribution of study sites CHC: Community Health Center; PHC: Primary Health Center

Facilities were selected based on a simple random sampling method from the list of all facilities. From all the community health centers (CHCs), four were randomly selected, and from all the selected CHCs, three primary health centers (PHCs) were randomly selected. From each PHC, two sub-centers were randomly selected for the study. From the list of private hospitals, seven private hospitals and seven private clinics were included in the study. The study district also contained one medical college and one district hospital, which were also included in the study (Figure 1). All selected facilities agreed to participate in the study.

Assessment method

A two-level assessment was done; first, at the level of facility and second, at the level of the individual health care staff. The facilities were assessed based on various identified domains and various identified sites according to the level of health care they were providing at the time of the study. The detailed data collection sites at each level of the facility are represented in Table 1.

**Table 1:** Sites assessed in different healthcare facilities SC: Sub-Center, PHC: Primary Health Center, CHC: Community Health Center, DH: District Hospital, MC: Medical College, PH: Private Hospital, PC: Private Clinic

	SC	PHC	CHC	DH	MC	PH	PC
IPD			YES	YES	YES	YES	
EMERGENCY		YES	YES	YES	YES	YES	YES
LR			YES	YES	YES	YES	
IMMUNIZATION ROOM	YES			YES	YES		
OT			YES	YES	YES	YES	
LABORATORY			YES	YES	YES	YES	

Assessment tool

A close-ended, semi-structured proforma was developed, validated, and used for eliciting data. Questions related to the following domains were asked to the participants: collection, segregation, storage, transportation and final disposal, awareness and practices regarding BMWM, guidelines, type of containers, polythene on containers, biohazard symbol, availability of lid on containers, availability of hub cutters, and presence of functional hub cutters and hazards related to BMWM.

Data collection

During the first visit to the facility, the investigator met the in-charge and identified the services being provided in the health facilities. Consent was taken, and a brief hospital review form was filled. If possible, the interview of the staff was done on that day itself. If not, then an appointment for an interview was taken for the next visit to the hospital. All in-charges of the concerned health care facility were interviewed to assess the current situation of biomedical waste management in the concerned health facility and the problems being faced by them in BMWM. A separate questionnaire was filled for other hospital staff to assess the knowledge, awareness, and health hazards associated with BMWM.

Ethical considerations

Before conducting the study, approval was taken from the institutional ethics committee (307/UPUMS/Dean/2017-18), and separate written consent was taken from the competent district authority. Written informed consent was taken from the participant on the participant's consent form after explaining the study objective and procedure as detailed on the participant information sheet.

Data analysis 

Data were entered in Excel (Microsoft Corporation, Redmond, WA). Internal consistency and validity of data were established by scrutiny of the data at the time of data entry and then by random re-checking after data entry. Data cleaning was done, and data were transferred to SPSS version 17 (SPSS Inc., Chicago, IL) for statistical analysis.

## Results

Evaluation of services

The total number of sites, like the emergency room, labor room, operation theater, pathology, dressing room, injection room, and immunization room, were observed. A total of 14, 33, 36, and 22 working sites were observed in tertiary, secondary, primary, and private facilities, respectively (Table 2).

**Table 2:** Sites observed in different healthcare facilities

S. No.	HCF	Sites						Total
		O.T	Wards	Labor Room	Laboratory	Immunization Room	Emergency	
1.	Medical College	4	6	1	1	1	1	14
2.	District Hospital	5	5	1	1	1	1	14
3.	Community Health Center	4	4	4	3	0	4	19
4.	Primary Health Center	0	0	0	0	0	12	12
5.	Sub-Center	-	-	-	-	24	-	24
6.	Private Hospital	2	2	2	2	0	7	15
7.	Private Clinic	0	0	0	0	0	7	7
8.	Total	15	17	8	7	26	32	105

**Figure 2:**
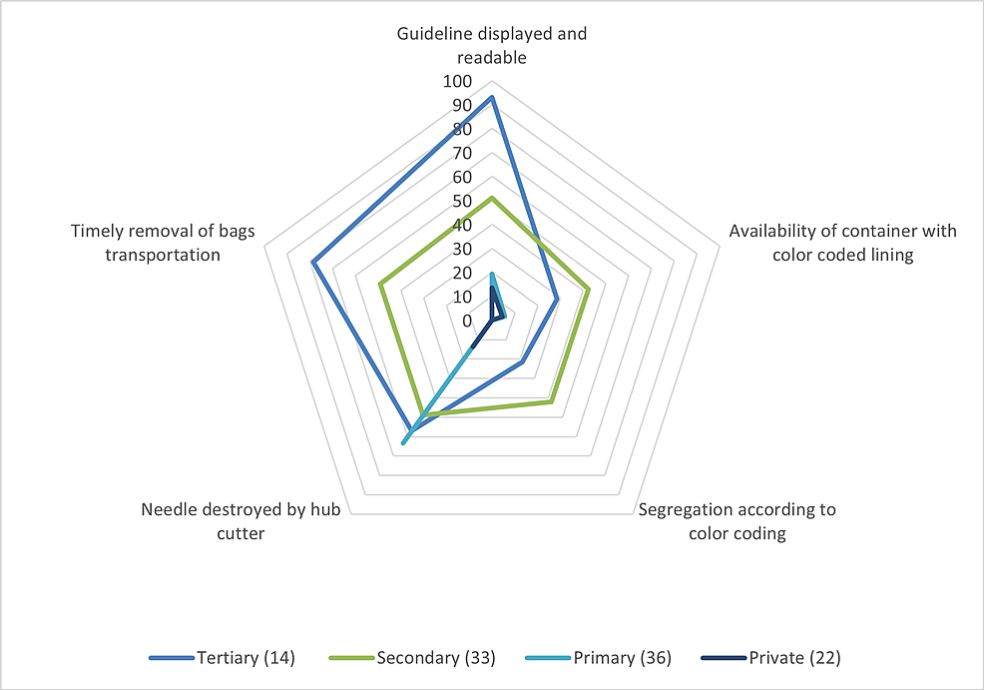
Distribution of observation according to the type of facility

Biomedical waste guidelines were mainly available at tertiary care centers (93%) and secondary care centers (51.5%). Availability of color-coded lining, segregation process, hub cutter, and timely transportation was not satisfactory at all types of health facilities. The tertiary care centers are better in terms of compliance with various points related to the BMWM, whereas the private facilities have lower compliance. However, the guidelines were displayed and there was timely removal of biomedical waste in the tertiary and secondary care facilities. All the above-mentioned points were lacking in primary care facilities and private facilities (Figure 2).

Health workers' awareness and preventive measures

Data related to awareness and preventive measures were collected from healthcare workers, including doctors and nurses working at facilities. These questions are categorized into two groups - general, that is, basic, questions, and specific, that is training and practice-related, questions (Tables 3-4).

**Table 3:** Awareness among health care providers (general) BMW: Biomedical Waste; BMWM: Biomedical Waste Management

S.N.	Awareness of Health Care Provider - General	Total Doctors (N=51)	Total Nurses (N=83)	P-Value
1.	Hazard associated with BMW?	51 (100%)	49 (59%)	<0.001
2.	Prevention of hazards associated with BMW?	51 (100%)	58 (69.8%)	<0.001
3.	Open unused sharps considered as BMW?	24 (47.0%)	25 (30.1%)	0.048
4.	Objects came in contact with blood or blood product considered as BMW?	27 (52.9%)	23 (27.7%)	0.003
5.	Content of Hazardous waste in BMW?	31 (60.7%)	15 (18.0%)	<0.001
6.	Should there be regular training regarding BMWM?	46 (90.1)	33 (39.7%)	<0.001
7.	Segregation reduces the cost of BMWM?	24 (47.0%)	19 (22.8%)	0.003
8.	Vehicles designated for transportation of BMW should not be used for other purposes?	36 (70.0%)	28 (33.7%)	<0.001
9.	Safe management of BMW is the responsibility of all?	24 (47.0%)	47 (56.6%)	0.281

**Table 4:** Awareness among Health Care Provider (Specific) GOI: Govt. of India; BMW: Biomedical Waste

SN.	Awareness of Health Care Provider -Specific	Total Doctors (N=51)	Total Nurses (N=83)	P Value
1.	Guidelines used by GOI for BMWM?	51 (100%)	66 (79.5%)	<0.001
2.	BMWM policy being followed in your HCF?	44 (86.2%)	25 (30.1%)	<0.001
3.	Color coding used for BMW?	31 (60.7%)	25 (30.1%)	<0.001
4.	Soiled waste segregated in?	34 (66.6%)	22 (26.5%)	<0.001
5.	Used sharps and needles segregated in?	27 (52.9%)	22 (26.5%)	0.002
6.	Glassware, ampules segregated in?	23 (45.0%)	13 (15.6%)	<0.001
7.	Reusable plastic material segregated in?	31 (60.7%)	23 (27.7)	<0.001
8.	Bio-hazard symbol	32 (62.7%)	33 (37.9%)	0.009
9.	Untreated BMW can store max to 48 hours?	10 (19.6%)	5 (6.02%)	0.015

Awareness of practices and their application is a very crucial step for biomedical waste management. Awareness among doctors related to hazards and prevention of hazards (<0.001), knowledge of unused sharp (0.048), contact with a blood-related product (0.003), hazardous waste, (<0.001), need for training (<0.001), awareness about segregation (0.003), and a separate vehicle for BMW management (<0.001) are statistically significant with respect to the nurses.

Health hazards related to biomedical waste management among doctors

Doctors and nurses both reported 12% needlestick injuries in the past 12 months (0.960). Nurses also recorded 8% other types of injury, which is significant (0.013).

Problems faced in implementing BMWM

**Figure 3:**
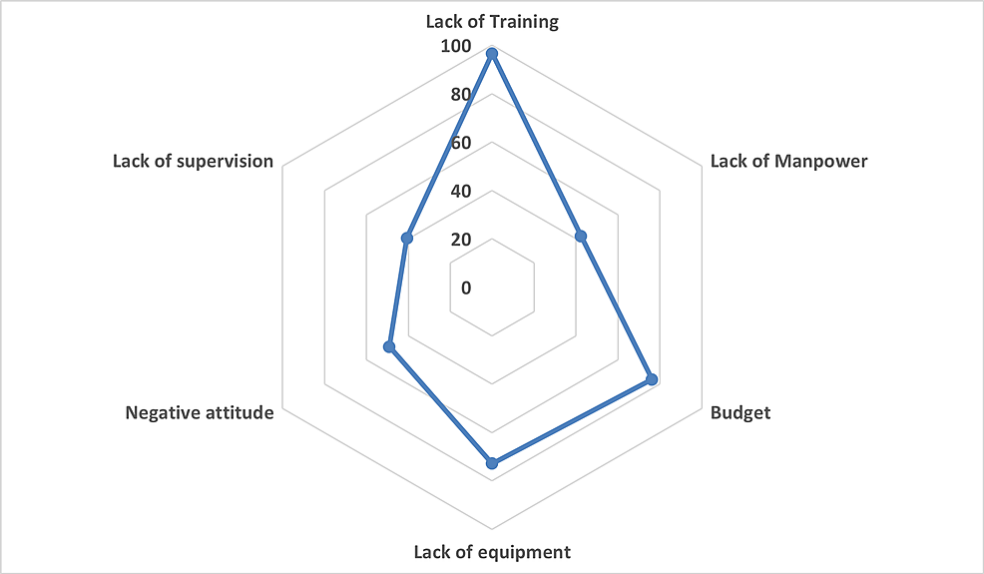
Problems faced by those in charge of facilities

People in charge of health facilities also faced several problems. Lack of regular training, inadequate budget, and logistics are the main bottlenecks to maintaining appropriate biomedical waste management. However, the lack of supervision, manpower, and negative attitude of the staff were not reported to be the problem by one-fifth of the health facilities (Figure 3).

## Discussion

The present study was carried out in 56 government and private health care facilities. Hospitals ranging from medical colleges to sub-centers were included from the government sector, while in the private sector, private hospitals and private clinics were included for the study. In the present study, most HCFs were from the government sector (75%), while the remaining health facilities were from the private sector (25%). A total of 105 sites were observed during the study period, including emergency rooms, immunization rooms, wards, operation theaters, labor rooms, and laboratories. We assessed the knowledge of BMW management among health care professionals, including 51 doctors and 85 nurses. Data regarding general information on health care facilities, awareness and existing practices, health hazards regarding BMW, and problems faced by facilities were collected from those in charge of dealing with BMW. We could not find any study similar to this evaluating services related to biomedical waste in India.

Awareness about biomedical waste management

Among 59 stakeholders interviewed, 45 were from government HCFs and 14 were from private HCFs. Guidelines laid down by the Government of India for BMWM suggest that every healthcare provider should be aware of BMWM, and, here, all the doctors (100%) and nurses (80%) were aware of BMW guidelines. Similar findings were observed in a study done by Narang RS et al., where all doctors (100%) were aware of prevailing BMWM guidelines [9]. In a study done by Pandit NB et al. and Sushma MK et al., 80% and 98% of doctors, respectively, were aware of BMWM guidelines [10-11]. These results were similar to the current study. In the present study, 86% of doctors and 30% of nurses were aware of BMWM policies being followed in their working healthcare facilities. This reported prevalence was more than that reported by Narang RS et al. and Rao et al. [9,12].

Health hazards

Doctors (12%) and nurses (8%) reported needle-stick injuries in the past 12 months, which is lower than that reported by Sharma et al. and Jayath et al. [13-14]. The differences may be due to the difference in the study site and time period of study. The inclusion of primary and community health facilities, which generally handle fewer emergency cases in the current study might have resulted in this lower prevalence of needle stick injuries.

Problems with implementation

In this study, the main problem cited with poor implementation was found to be the non-availability of trained staff followed by a lack of equipment and the unavailability of budget to procure the same. The lack of supervision and lack of manpower was the least cited reason. The above-mentioned finding is against the previous finding. This might be due to the less trained sanitation staff in the facilities. This is similar to that reported by Lohani et al. [15].

## Conclusions

The study is one of the few studies that included health facilities, including the sub-centers, PHC, and CHCs. Non-segregation of waste at the point of generation and the nonavailability of color-coded bins were prevalent in all types of facilities. The knowledge of the health care workers was found to be satisfactory. The problems listed were lack of training and lack of availability of budget regarding the same. All these lacunae are more or less preventable, related to a lack of willpower by the competent authority. Effective monitoring and training of the sanitation staff might help in the appropriate management of biomedical waste.

## Appendices

A descriptive study on bio-medical waste management in health care facilities of Etawah district.        

1.      Is guidelines or chart for BMW displayed:            yes/no      If yes,

2.      Location of chart:               appropriate/ inappropriate

3.      Content readable:                yes/no

4.      What kind of container present-  no specific/ plastic/metal/ cardboard/ others?

5.      Is yellow color container available        yes/no         If yes-

6.      Has yellow bag been placed lining the inner side of yellow container?    yes/no

7.      Has biohazard symbol imprinted on yellow containers?    yes/no

8.      Does yellow bag contain infected, soiled waste?         yes/no

9.      Is red color container available              yes/no     If yes

10.  Has red bag been placed lining the inner side of red container?          yes/no

11.   Has biohazard symbol imprinted on red bag or container?     yes/no

12.  Does red bag contain only plastic waste?      yes/no

13.   Is blue color container available            yes/no      If yes,

14.   Has blue bag been placed lining the inner side of blue container?      yes/no

15.  Has biohazard symbol imprinted on blue bag or container?     yes/no

16.  Does blue bag contain only sharp waste?       yes/no

17.   Is white translucent puncture proof container available ?        yes/no  If yes

18.   Does white puncture proof, translucent container contain only for sharp waste           yes/no

19.  Has bio hazard symbol imprinted on white container or bag       yes/no

20.  Has containers covered?             yes/no

Needle Handling Practices

21.  Hub cutter / needle destroyer present?        Yes/no

22.  If present, functional            yes/no

23.  Used needles destroyed?          yes/no

24.  Are used needle found recapped?        yes/no

25.  Are nozzle of used syringe destroyed?       yes/no

26.  Plunger placed in red bag?       Yes/no

27.  Are needles bend manually?               Yes/no

       In house transportation and storage at facility level:

1.      Are bags removed before 3/4th had full?       yes/no

2.      Frequency of removal of waste?     Once daily/ twice daily/ alternative day/……….

3.      Trolley or equipment for transport available?     Yes/no

4.      Specific waste storage area available?         Yes/no

5.      Is it secure?           Yes/no

6.      Bags transported from point of generation to storage area?         Trolley/manually/others

7.      Weighing machine available?     Yes/no

8.      Area only accessible to authorized person?         Yes /no

9.      Log book available            yes/no

10.  Log book updated              yes/no

11.  Storage time for untreated BMW           <48 hrs./ >48h

Treatment or Disposal 

1.      Treatment or disposal             on site/ offsite

        If on site ,

2.      Which equipment are available              incinerator/ microwave/ autoclave/shredder/….

3.      Final disposal    burning/deep burial/ open dumping/…….

4.      Pretreatment done?   yes/no

5.      Chemical used for the pre-treatment of biomedical waste.

6.      Type of waste management facilities available

a) Collection     yes/no

b) Segregation   yes/no

c) Storage   yes/no

d) Transportation yes/no

1.      Are there any guidelines by GOI for BMW management?          

   a) Yes           b) No             

2.      Is there any biomedical waste management policy in your hospital/clinic? 

a) Yes          b) No            

3.      Knowledge about the different colour coding used by GOI for segregation of Bio-Medical Waste in the past?

  a) Yes              b) No                

4.      Knowledge about soiled dressings, human anatomical waste are segregated in?

a) Yes       b ) No                

5.      Knowledge about used sharps and needles are segregated in?

a) Yes              b) No                 

6.      Knowledge about glassware, ampoules are segregated in?

7.       a) Yes              b) No                

8.      Knowledge about reusable plastic material segregated in?

a) Yes              b) No                

9.      Knowledge about the bio-hazard symbol displayed on the containers?

a) Yes              b) No                

10.  Knowledge about the hazards associated with poor management and handling of Bio-Medical Waste?    a) Yes              b) No                

11.  How can prevent hazards associated with poor management and handling of Bio-Medical Waste?  a) Yes              b) No                

12.  Open unused sharps are considered as bio-medical waste?

a) Yes              b) No                

13.  Any item which has had contact with blood or any other body fluid is considered as bio-medical waste?

a) Yes              b) No                

14.  Untreated bio-medical waste can be stored maximum for 48 hours?

a) Yes              b) No                

15.  About 10-15% of total waste generated in a hospital is hazardous?

 a) Yes              b) No                

16.  Safe management of biomedical waste is the responsibility of

a) only government   b) Doctors, nurses, paramedics   

17.  Should there be regular training biomedical waste management? 

a) Yes              b) No               

18.  Proper segregation reduced the cost of BMWM?

a) Yes              b) No                

19.  Vehicle designated for transportation of BMW should not be used for other purpose use?   

a) Yes              b) No              

HCF In-Charge/ Hospital Manager/ Biomedical Waste In-Charge

1.      Specific post for HCWM in your facility? Yes/No

2.      What are the problems you are facing in implementation of waste management?

·         Lack of training of staff 

·         Lack of Manpower

·         Poor compliance of common biomedical Waste treatment facility(CBWTF)

·         Budget constraints

·         Lack of equipment

·         Negative attitude of health care personnel

·         Lack of supervision

Health Hazard

1-      Are you faced any needle stick injury in past 12 months?   Yes/no

2-      If yes, what measures do you take when it happens?  Reported/not reported

3-      Are you vaccinated against Tetanus?  Yes/No

4-      Are you vaccinated against Hepatitis B?  Yes/No

5-      Are you using protective measures?  Yes/No

6-      Are you have taken any training or sensitization on waste handling? Yes/no
